# Cancer-Related Stress and Complementary and Alternative Medicine: A Review

**DOI:** 10.1155/2012/979213

**Published:** 2012-07-15

**Authors:** Kavita D. Chandwani, Julie L. Ryan, Luke J. Peppone, Michelle M. Janelsins, Lisa K. Sprod, Katie Devine, Lara Trevino, Jennifer Gewandter, Gary R. Morrow, Karen M. Mustian

**Affiliations:** James P. Wilmot Cancer Center, Department of Radiation Oncology, School of Medicine and Dentistry, University of Rochester Medical Center, Saunders Research Building, 265 Crittenden Boulevard, Office 2.224, Box CU 420658, Rochester, NY 14642, USA

## Abstract

A cancer diagnosis elicits strong psychophysiological reactions that characterize stress. Stress is experienced by all patients but is usually not discussed during patient-healthcare professional interaction; thus underdiagnosed, very few are referred to support services. The prevalence of CAM use in patients with history of cancer is growing. The purpose of the paper is to review the aspects of cancer-related stress and interventions of commonly used complementary and alternative techniques/products for amelioration of cancer-related stress. Feasibility of intervention of several CAM techniques and products commonly used by cancer patients and survivors has been established in some cancer populations. Efficacy of some CAM techniques and products in reducing stress has been documented as well as stress-related symptoms in patients with cancer such as mindfulness-based stress reduction, yoga, Tai Chi Chuan, acupuncture, energy-based techniques, and physical activity. Much of the research limitations include small study samples and variety of intervention length and content. Efficacy and safety of many CAM techniques and some herbs and vitamin B and D supplements need to be confirmed in further studies using scientific methodology. Several complementary and alternative medicine therapies could be integrated into standard cancer care to ameliorate cancer-related stress.

## 1. Introduction 

Cancer-related distress is defined as an “unpleasant emotional experience of a psychological, social, and/or spiritual nature that may interfere with the ability to cope effectively with cancer, its physical symptoms, and its treatment” [1]. Several factors can cause stress during the cancer experience; a cancer diagnosis itself is a strong stressor associated with “disbelief, anxiety, depression,” and disturbances of sleep, appetite, and routine daily activities [2]. In addition to uncertainty about the disease and its treatment, there is also fear of death, disease progression, reduction in quality of life (QOL) and relationships, a loss of sense of control [3–10], and impacts on decision-making ability and treatment compliance [1]. Cancer patients experience a broad spectrum of individual and cooccurring symptoms such as pain, anxiety, depression, fatigue, nausea, diarrhea, wasting, and cognitive impairments, which both promote and indicate distress [11]. Regardless of treatment regimen, distressing symptoms such as fatigue, insomnia, pain, depression, hot flashes, sexual dysfunction, and cognitive deficits frequently occur and often persist following treatment [12, 13]. Overall, a cancer diagnosis creates a vicious and compounding cycle of stress. 

Although all patients with a history of cancer experience variable level of stress across the continuum of disease [1], often information sharing on this topic does not happen during interaction of patients with their healthcare professionals [1]. The reported prevalence of cancer-related distress is 24–59% depending on the type of cancer [10], stage of disease, patient population studied, and study setting [14, 15]. A recent study of newly diagnosed cases reported distress in about 67% of lung cancer patients and 50% of breast cancer patients [16]. Another study found self-reported distress in 25% of cancer outpatients, 59% of patients with advanced cancer undergoing palliative care, and 16% of cancer patients in the general community [14]. Psychosocial interventions including experiential-existential group psychotherapy and cognitive-behavioral stress management [17–22] have shown positive results in coping with daily stressors. Additionally, pharmacologic treatments for some of the cancer-related psychiatric symptoms are available. Some resources are available in the form of information on its identification and possible counseling services recommended by various national societies and institutions [1]. However, only a small percentage of patients with distress are detected and referred for treatment [1]. The use of complementary and alternative medicine (CAM) has increased among cancer patients at the time of diagnosis, during treatment, and even after treatment is complete [23]. The primary reasons for CAM use by cancer patients are pain relief, immune-system boost, symptom management [24], and better quality of life [25]. However, there are concerns about the use of CAM techniques related to cancer experience since the efficacy of several of these techniques/products has not been documented or due to possibility of interaction with treatment. This paper reviews aspects of cancer-related stress and CAM interventions for the amelioration of stress during and after the cancer experience. Researchers over time have used the words “stress” and “distress” interchangeably, and in this paper, the term “stress” will be used unless the referenced study used the word “distress”. 

## 2. Neuroendocrine and Immunological ****Aspects of Stress and Cancer

**Figure 1:**
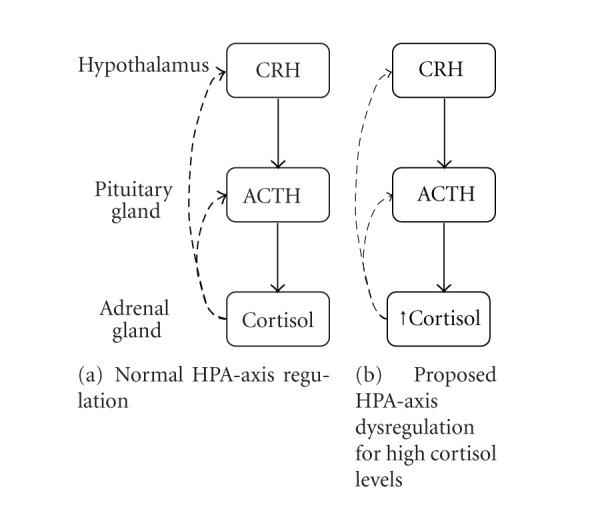
Overview of the hypothalamus-pituitary-adrenal axis in relation to the stress response in healthy individuals (a) and those with cancer (b). CRH: corticotrophin-releasing hormone; ACTH: adrenocorticotropic hormone. Thin dashed lines represent reduced feedback inhibition.

Stress is characterized by psychophysiological processes in response to an event or circumstance that is perceived as threatening, harmful, or challenging [26]. The hypothalamus-pituitary-adrenal (HPA) axis and the sympathetic nervous system (SNS) are involved in the physical stress response. The HPA axis functions through a negative feedback system: increased cortisol and other glucocorticoid (GC) levels inhibit release of corticotrophin-releasing hormone (CRH) and adrenocorticotropic hormone (ACTH) from the neurons of the hypothalamus and pituitary gland, respectively, leading to a reduction in GC levels (Figure 1(a)). A chronic or repeated exposure to a stressor decreases CRH, ACTH, and GC levels [27, 28] indicating a reduction in negative feedback in the HPA axis. In cancer patients, such dysregulation of the HPA axis and the SNS may be related to the development, maintenance, and recurrence of cancer. For instance, norepinephrine has been linked to the etiology of cancer [29], and epinephrine has been shown to protect against cellular apoptosis in cancer cells [30]. In women with metastatic breast cancer, HPA axis dysregulation was characterized by increased resting cortisol levels (Figure 1(b)) and decreased inhibition of the pituitary gland and the hypothalamus [31]. Although a linear relationship between resting cortisol levels and stage of cancer was observed, ACTH levels were normal in both early and late stages [31]. Other studies have shown that some cancer patients have a flattened diurnal cortisol rhythm compared to healthy controls [32, 33], which is associated with shorter survival times [33]. 

Stressful events like cancer have been shown to lead to increased risk of disease progression and decreased survival [34–37], and they can contribute to dysregulation of the immune system, chronic inflammation, and numerous adverse effects [38]. Psychological stress and altered HPA axis function can influence the activity of a variety of immune cells, including natural killer (NK) cells, T cells, and macrophages [39]. Disrupting the balance of immune cells leads to a chronic proinflammatory cytokine-mediated cascade of events resulting in enhanced psychological stress, depression, anxiety, fatigue, sleep disturbance, cognitive impairments, and ultimately reduced quality of life [40–44]. Stress hormones can impair the immune response and may affect tumor progression and cancer prognosis. Chronically elevated stress hormones shift the balance between the Th1 (cellular) and Th2 (humoral) immune responses toward the Th2 response. Expression of Th1 cytokines IFN-*γ* and IL-12 decreases during stress, while Th2 cytokines IL-4, IL-5, IL-6, IL-10, and IL-13 increase (reviewed in [45, 46]). These changes are associated with decreased cytotoxic T-lymphocyte and natural killer (NK) cell activity [47, 48]. Evidence from animal models suggests that these types of immune deficiencies can lead to tumor progression. For example, mice subjected to social isolation stress had decreased splenic NK cell activity [49]. In the same mice, metastasis required less time after tumor cell injection in stressed animals than in controls. Stressed animals also did not respond as well to chemotherapy [50]. 

Stress hormones can also alter cell-signaling pathways, which have been implicated in cancer progression. Many studies suggest that stress hormones can decrease apoptosis in certain cancer cells through decreased activity of pro-apoptotic caspases-3, 8, and 9 [51] and protein BAD [52]. Increases in vascular endothelial growth factor (VEGF), which is important for tumor vascularization and survival [53], have been shown in cancer cell culture models as a result of norepinephrine-dependent *β*-adrenoreceptor activation of the cAMP/PKA signaling pathway [54–56], and this could be critical in tumor progression. In an animal model of ovarian cancer, psychological stress simultaneously increased VEGF expression and tumor burden [57]. 

Metastasis relies on tumor cell invasion, which requires proteins that can break down the extracellular matrices of the invaded tissues; preliminary evidence of the same is provided by norepinephrine-stimulated ovarian cell invasion and increased matrix-metalloproteinase- (MMP-)2 and 9 expression through activation of the *β*-adrenergic receptor [58] and an increase in MMP-2 and 9 associated with psychological stress in the ovarian cancer mouse model [57]. Thus, it is conceivable that stress management might reduce disease progression and improve quality of life in patients with cancer. We note that while this is a plausible hypothesis, the evidence supporting it in cancer patients is lacking. Several modalities for stress reduction have shown promise; use of other options can be considered based on evidence provided by recent research on complementary and alternative medicine (CAM) techniques/products. 

## 3. Complementary and Alternative Medicine**** Utilization in Cancer

CAM has been defined by the National Center for Complementary and Alternative Medicine (NCCAM) as “a group of diverse medical and health care systems, practices, and products that are not generally considered part of conventional medicine” [59]. Such techniques include mind-body medicine, natural products, nutritional supplementation, manipulative body-based practices, energy-based techniques, and traditional medical systems (e.g. Chinese, Ayurved, and American-Indian). These modalities have recently been classified as integrative medicine as they are not an alternative to conventional medicine use; however, for the purpose of this paper the term CAM will be employed. CAM use has been increasing among cancer patients [23] and currently ranges from 22% to 73% [60]. Based on 2002 National Health Interview data, 26% of female survivors and 13.7% of male cancer survivors reported using CAM [61]. A more recent study revealed that 62% of female breast cancer survivors used CAM [62]. CAM use in cancer patients is associated with high levels of distress and failure of conventional medicine to meet psychosocial needs [63]. Although emotional distress along with other psychological indices significantly predicted CAM use in male and female survivors of colon cancer [64], a study of predictors of CAM use in early-stage breast cancer within a year of diagnosis suggested that such use may be motivated by the expected benefits and may not necessarily indicate distress or dissatisfaction [65].

## 4. Mind-Body Medicine

Mind-body medicine (MBM) includes a variety of practices that enable the mind to influence body functions. Some of the early works in the area of MBM include research on transcendental meditation (TM) [66–69] and application of meditation techniques for stress reduction by Kabat-Zinn [70]; the latter was called mindfulness-based stress reduction (MBSR). MBM is increasingly used by breast cancer patients [71]. A recent study reported that 64.2% of breast cancer patients practiced mind-body techniques following their diagnosis, and this usage was associated with Hispanic race, higher education, low income, and other CAM use [72]. Research on MBM in cancer has grown in the past decade, although the majority of studies have been conducted in the breast cancer population. Moreover, studies have used a variety of designs, intervention programs, and measures to evaluate effects, and most of the research has involved small sample sizes. Larger studies are needed to confirm the effects of many mind-body techniques on common symptoms of cancer and its treatment. 

Several studies in cancer patients have examined the effect of meditation and yoga on quality of life, fatigue, and sleep [73–80]. Mindfulness-based stress reduction (MBSR), a program that includes meditation, yoga postures, and relaxation [81], helps patients understand their personal responses to stress and teaches them how to modify their responses. A program of MBSR reported lower levels of total mood disturbance and distress [82] as well as significant improvements in mood, sleep quality, and fatigue in a mixed cancer population [83]. Other studies using MBSR interventions in cancer patients have shown decreases in stress symptoms and cortisol levels and improvements in patient-reported quality of life [74]. A recent randomized controlled trial of a 6-week MBSR program for breast cancer survivors reported reduced anxiety, depression, and fear of recurrence and better perception of physical functioning in the intervention group [78].

Sixteen studies have shown that participation in programs consisting of traditional holistic yoga results in statistically significant improvements in stress, anxiety, irritability, emotional well-being, sadness, energy, invigoration, cognitive function, relaxation, pain, sleep, mood, depression, fatigue, symptom severity, hot flashes, appetite, bowel function, nausea, vomiting, QOL, and tolerance of cancer treatment [73, 75, 76, 84–96]. The yoga interventions typically lasted from 6 to 24 weeks and most often involved weekly sessions of 75–120 minutes. The types of yoga used in the interventions also varied; the majority of the interventions used systems of yoga that involved meditation, breathing, gentle yoga, and restorative yoga. These studies indicate that clinicians and patients are very receptive to stress reduction programs, including yoga, as a treatment modality in traditional cancer centers and that it is feasible to recruit patients and conduct these types of interventions in a wide variety of communities. A large community-based trial of a yoga program in cancer survivors was found to improve their sleep, fatigue, quality of life [97], circadian rhythm, anxiety, and mood [98]. Some studies of yoga have examined its effects on measures of stress and its physiologic parameters. Yogic relaxation training has been found to reduce perceived stress after surgery in breast cancer [99]; another study of yoga during radiation treatment in breast cancer patients observed a 27% reduction in perceived stress score [84]. A recent study of Iyengar yoga in breast cancer survivors observed reduced morning and evening cortisol levels along with improved fatigue, emotional well-being [73], and vitality and reduced pain [96] following eight- and twelve-week interventions.

Tai Chi Chuan (TCC) is a form of martial arts used for centuries in China as a health exercise involving a series of individual movements continuously linked together and performed in conjunction with deep breathing and mental concentration. At least 20 prospective, randomized, controlled clinical trials in a number of populations including the elderly, cardiovascular patients, and patients with chronic diseases have been conducted using TCC [100]. TCC as an intervention may provide benefits to cancer survivors related to physical deconditioning, cardiovascular disease risk, and psychological stress. In a randomized, controlled clinical trial conducted by Mustian et al., women who completed treatment for breast cancer and received TCC demonstrated significant improvements in functional capacity, aerobic capacity, muscular strength and flexibility, self-esteem, bone health, immune function, and QOL [101–106]. Thus, physical activity seems to be an intervention capable of reducing anxiety and distress associated with the cancer experience. Conversely, higher levels of anxiety may reduce the likelihood of participation in physical activity following cancer treatment. 

Acupuncture, a mind-body technique that is also classified as manipulative body-based technique and energy-based technique, has been shown to ameliorate distress in healthy adults [107]. It has also been found to reduce fatigue and distress in patients with advanced breast and ovarian cancer [108]. A recent systematic review of 15 studies of CAM interventions (acupuncture, massage, yoga and relaxation, hypnosis, vitamins, and medical qigong) in cancer-related fatigue reported most benefits from acupuncture [109]. Acupuncture has also been found beneficial in cancer-related vasomotor symptoms [110] and anxiety associated with hot flashes [111] and other symptoms associated with cancer such as pain, nausea and vomiting, fatigue that could be related to stress [112]. Another MBM technique, hypnosis, combined with cognitive behavioral therapy prevented the increase of fatigue in breast cancer patients compared to standard medical care during radiation therapy [113] and reduced fatigue in women who underwent lumpectomy for breast cancer [114]. 

## 5. Herbal and Natural Products

The use of herbal and natural supplements has dramatically increased over the last ten years. Herbal (or natural) supplements are commonly used to combat stress-related symptoms such as anxiety, depression, insomnia, and fatigue [115–117]. Herbal supplements are one of the three most common forms of CAM used by cancer patients [116–119]. In a recent study conducted by MD Anderson Cancer Center, 52% of 309 cancer patients reported using one or more forms of CAM modalities, and 26% reported herbal supplement usage [118]. Unfortunately, little evidence supports the effectiveness of herbal interventions for long-term reduction in stress [115, 116]. 

**Figure 2:**
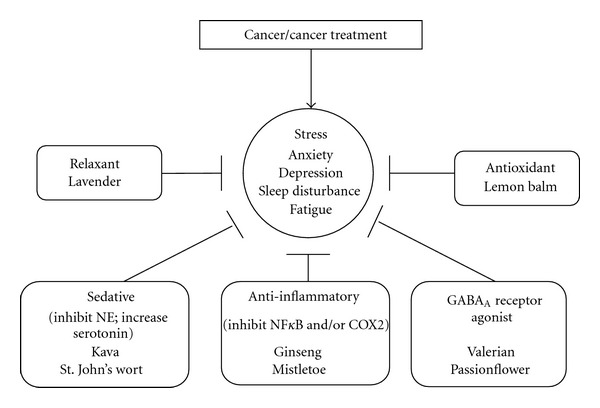
Mechanisms of herbal supplements for stress. Cancer and cancer treatment affect a patient's quality of life resulting in various symptoms of stress, such as anxiety, depression, sleep disturbance, and fatigue [155]. The human body's response to such stressors involves many different mechanistic pathways. This diagram outlines the mechanisms by which herbal supplements reduce stress-related symptoms in cancer patients. The majority of herbal supplements have anti-inflammatory properties, but their primary target of action is different. Lavender acts as vascular smooth muscle relaxant through nitric oxide/cGMP phosphorylation and myosin light chain dephosphorylation [156]. Lemon balm is an immune stimulating agent with potent free radical scavenging properties [157–159]. Kava and St. Johns's wort function reduce norepinephrine (NE) and increase serotonin levels, similar to antianxiety drugs such as benzodiazepines [116, 122, 160]. Valerian and passionflower are GABA_A_ receptor agonists that produce a sedative effect [161, 162]. Ginseng and mistletoe are potent anti-inflammatory agents that inhibit NFkB and/or COX2 [127, 163].

Sedative herbal and natural supplements have been used since the middle ages to reduce stress and improve quality of life. Herbal supplements are usually ingested as extracts (i.e., tea) or capsules or inhaled as essential oils (i.e., aromatherapy). Commonly used herbal supplements for stress include lemon balm, kava, valerian root, lavender, St. John's wort, and passionflower [115–117]. Lemon balm has proven effective and safe for relieving stress with long-term use [116, 120–123]. Substantial evidence indicates that kava reduces anxiety and stress; however, it has been implicated in liver failure and is therefore not clinically recommended but may be safe for short-term use in patients with mild to moderate anxiety [115, 116, 119, 122, 124, 125]. Although valerian is considered safe at low doses for less than one month, no clinical evidence supports its use for anxiety or distress [116, 121–123, 125]; however, combined with lemon balm and kava it has been associated with reduction in stress-related insomnia [120, 121, 125]. Lavender aromatherapy is recommended to relieve anxiety and depression and promote calmness and positivity, but definitive evidence for efficacy is lacking [117, 123, 124]. A randomized controlled trial which combined lavender essential oils with massage in patients with cancer reported reductions in distress, but the study was not able to conclude whether lavender aromatherapy supplemented the effects of massage [124]. Likewise, passionflower demonstrated a reduction in anxiety compared to oxazepam [116, 126]. St. John's wort is used mostly for depression and is less popular for treating anxiety and distress [116]. Some evidence suggests that Siberian ginseng and European mistletoe may reduce side effects of cancer treatment and improve quality of life [127–129]. Possible mechanisms of some herbals in stress are depicted in Figure 2. Thus patients with high levels of stress could benefit from herbal supplements [116–118, 124] but patients should discuss any proposed supplement use with their physician to ensure safety. 

Researchers have studied the effects of numerous vitamins, minerals, and dietary supplements on psychological stress. The most promising include the B vitamins (folic acid, B6 and B12) and vitamin D. However, there is a dearth of investigation of the effects of these compounds on psychological distress in cancer patients, perhaps due to the reluctance of oncologists to prescribe vitamin and mineral supplementation during treatment, believing that the antioxidant effects of these supplements might decrease treatment efficacy. Nevertheless, the few studies available show that vitamin and mineral supplementation administered during treatment do not reduce treatment efficacy [130, 131]. Following the completion of treatment, cancer survivors use vitamin and mineral supplementation at a higher rate than the general population, with 64–81% of cancer survivors using supplements compared to approximately 50% of the general population [132].

A large amount of the literature reports the effects of B vitamins (folic acid (B9), pyridoxine (B6), and cobalamins (B12)) on psychological stress, particularly depression. There is a biological rationale for the association of B vitamins with psychological stress. B vitamin deficiency can lead to an increase in homocysteine levels [133], which is associated with increased depression rates [134]. Cross-sectional studies commonly find that patients with psychological stress disorders have deficient folic acid levels [135–137]. Similar findings were reported for vitamin B12, with low levels seen in patients with psychological distress [138–140]. Evidence from cross-sectional studies also indicates that psychologically stressed patients have low levels of vitamin B6 [141, 142], but the evidence is not as strong as for folic acid or vitamin B12. Other studies show that individuals deficient in B vitamins have a poorer response to antidepression therapy and higher rates of relapse of depression. Randomized trials have shown that adding folic acid and vitamin B12 supplementation to existing treatments increases the efficacy of antidepressant treatment [143–145].

Recent research has shown that vitamin D may be involved in psychological well-being. Vitamin D plays a crucial role in brain development and function [146, 147]. A recent study in fibromyalgia patients found a significantly higher rate of vitamin D deficiency in patients with anxiety [148]. Other studies have associated vitamin D deficiency with cognitive impairment [149, 150], mood disorders [150], and depression [148, 151]. Vitamin D deficiency has also been associated with seasonal affective disorder (SAD), a condition with depression-like symptoms that occur in winter, when vitamin D levels are typically at their lowest [152]. Randomized trials also show that vitamin D supplementation may ameliorate symptoms of depression [151] and SAD [153, 154]. Thus, research indicates that individuals with low levels of folic acid, vitamin B12, and vitamin D have higher rates of psychological stress, and limited evidence from randomized trials show that supplementation with these vitamins may improve anxiety, mood disorders, and depression. 

## 6. Manipulative and Other CAM Therapies 

Research on massage therapy, a manipulative body-based technique, for stress reduction in cancer populations has not provided consistent results [164–167]. Polarity Therapy, Reiki, therapeutic touch, healing touch, and Qigong involve manipulation of the body energy fields. Polarity therapy (PT) has been shown to reduce cancer-related fatigue during radiation treatment [168] when compared with modified massage therapy and standard care [169]; the potential for its use in management of cancer-related stress can be explored since PT has been shown to decrease stress in caregivers of dementia patients [170]. The fact that Reiki ameliorates pain in advanced cancer patients [171] and reduces cancer-related fatigue [172] may indicate that it can also lower cancer-related stress, although there is no supporting evidence. The trials of music in reducing stress in cancer have not yielded consistent results; one study in women with metastatic breast cancer showed no significant differences in the music therapy led by a therapist and the usual care groups [173]. However, one study of music imagery intervention has suggested reduction of anxiety in adults undergoing chemotherapy, particularly those with lower initial stress levels [174]. 

## 7. Exercise

Exercise training improves resilience to stress [175, 176]. A 6-week exercise intervention in patients receiving chemotherapy was found to have beneficial effects on psychological distress [177]. Similarly, in postoperative breast cancer patients receiving chemotherapy, a 12-week home-based exercise intervention was found to improve mood disturbance compared to a nonexercise control group [178]. Researchers have found that cancer patients who exercised before undergoing treatment for cancer experienced lower levels of anxiety and depression [179]. Early-stage breast cancer patients undergoing a 6-week walking exercise intervention during radiation treatment also noted improvements in anxiety [180]. In colorectal cancer survivors, a relationship was established between psychological distress, anxiety, and participation in physical activity. Cancer survivors with higher levels of anxiety are less likely to participate in physical activity [181]. 

## 8. CAM in Children, Adolescents, and ****the Elderly with Cancer

A recent systematic review estimated that the prevalence of CAM use by children with cancer ranges from 6% to 91%, with significant variation between studies [182]. Children's and adolescents' use of CAM has been linked to parents' use of CAM [183, 184]. Parents with higher educational backgrounds are more likely to consider [185] and to use [182] CAM approaches for their children, although no consistent correlations between CAM use and parental income, child age, or ethnicity have been reported [182]. CAM use and consideration were shown to be positively related to a lower survival perspective [186] and fewer days since relapse [185], respectively, suggesting that CAM therapies may be a coping strategy for families in an attempt to try every possible approach for curing or alleviating pain and distress in their children. A review by Sencer and Kelly [187] suggests that families' use of CAM may increase their sense of control and active participation in treatment, and therefore open discussion of CAM should be useful for physicians in building relationships with families and in understanding all potential influences on the child's care.

The quality of research related to CAM use in pediatric oncology has varied greatly from study to study. Within the past 10 years, there has been considerable movement towards more rigorous research testing, including randomized clinical trials conducted through the Children's Oncology Group or other multisite studies [188–191]. There is a great need for rigorous safety and efficacy trials, particularly for biologically based therapies [188, 192]. Research has reported physical and psychological benefits from a massage intervention for children with various cancers and blood disorders [193]; however, another randomized trial of a combined massage and humor intervention found no significant differences across groups in children undergoing stem cell transplants [190]. A trial of music video compared to audiotape and usual care in adolescents and young adults undergoing stem-cell transplantation suggested less distress in music group at 100-day follow-up [194]. Thus, several promising CAM therapies are available to help children with cancer to manage emotional and physical distress related to cancer and its treatment; however, much work is needed to document their efficacy and safety.

Aging alone is associated with declines in physical function, including reduction in functional capacity [195, 196], reduced muscular strength [197], arthralgia [198], and reductions in bone mineral density [199]. Depression [200], anxiety [201], and cognitive difficulties [202] also affect older adults. Cancer treatments can further exacerbate these age-related declines and stressors. Cancer survivors aged 65 and older report more limitations in activities of daily living [203], a lower quality of life [204], lower self-rated health [204], a greater incidence of frailty [203], and higher rates of dementia, depression, falls, incontinence, and osteoporosis [203]. Other stressors in this population include relocation, financial changes with retirement, caring for grandchildren and/or a spouse, the death of friends and family members, chronic illnesses, and the fear of losing independence [205–208]. Stress is associated with depression in the elderly [209]. Emotional stress is also a potential trigger impairing the mechanisms responsible for balance, increasing the risk of falls that can lead to hip and femur fractures [210]. These additional declines in physiological and psychological function likely exacerbate the stress experienced by older cancer survivors. 

Physical activity and social interactions reduce functional decline in the elderly [211]. Physical activity is also associated with a reduction in stress and anxiety. The American College of Sports Medicine (ACSM) recommends that older adults, even those with chronic medical conditions, participate in regular aerobic exercise training (150–300 minutes per week) and resistance exercise training (at least 2 days a week) [212]. The exercise prescription for those older adults who are functionally limited should be tailored and progressed gradually [213]. The literature supports the use of physical activity in cancer survivors younger than 65, but little has been done to determine the benefits of physical activity or other forms of complementary and alternative medicine for cancer survivors who are 65 years of age and older. Researchers have investigated the benefits of Tai Chi Chuan in older adults without a history of cancer, finding that Tai Chi can reduce depression [214], improve self-efficacy [215], improve muscular strength and endurance [216], and improve balance and reduce falls [217]. Yoga can improve gait [218], quality of life [219], and depression [220] in the elderly. Research is limited concerning the implementation of CAM in older cancer survivors in particular, despite its promising impact on physiological and psychological function. Stress in particular has received very little research focus in older cancer survivors, but because the benefits of physical activity are so profound in older adults, it is an important and promising area of research. 

## 9. Conclusions and Future Directions

Cancer-related stress affects all patients with cancer and negatively impacts cancer outcomes in terms of response to treatment, quality of life, disease progression, and survival in different phases of their experience. Feasibility of intervention with several CAM techniques and products commonly used by cancer patients and survivors has been established in some cancer populations: for example, mind-body techniques of meditation, yoga, Tai Chi Chuan, acupuncture, manipulative techniques massage, energy-based polarity therapy and Reiki, and some natural products. Efficacy of some CAM techniques and products in reducing stress and/or stress-related symptoms in patients with cancer has been documented

1. Mindfulness-based stress reduction program with components similar to yoga showed reduction of stress levels in population of breast cancer and prostate cancer patients and improvements in endocrine indices of stress were also reported. Some studies of yoga intervention have shown significant stress reduction while some have shown beneficial effects on symptoms associated with stress for example, fatigue, sleep disturbances, hot flashes, and quality of life. However, the majority of studies of yoga were conducted in small samples of patients and there was a wide range of the length of intervention. This area of mind-body intervention seems to be promising in cancer; however, lack of uniformity of the intervention program in terms of its length and content makes it difficult to compare study results. Larger study samples with the use of comparable intervention programs may be more conclusive.
2. Acupuncture can relieve anxiety, fatigue, and distress associated with advanced cancer.
3. Practice of Tai Chi Chuan may be helpful in improving quality of life.
4. Some herbs like lemon balm may be used, long in the term for relieving stress. Most current research suggests that patients with high levels of stress benefit the most from herbal supplements; therefore, studies are needed to examine their efficacy as well as safety.
5. Most research on vitamins and supplements conducted in noncancer patients show promise in relieving stress; further trials in cancer patients are needed to demonstrate the safety of vitamin B and D supplementation in cancer patients receiving treatment before testing their efficacy on psychological stress.
6. Physical activity may be helpful in reducing anxiety and distress in cancer survivors; however, the role of anxiety in affecting physical activity should be given consideration while designing intervention studies in cancer population.
7. Research on CAM therapies for stress in childhood cancers is insufficient. Several promising CAM therapies may help children with cancer manage emotional and physical distress related to cancer and its treatment after the safety and utility of such therapies has been established.
8. Stress in older cancer survivors is an important and promising area that is desperately needed to be examined as older adults are at much greater risk of developing cancer than young adults. It is especially important since by the year 2030, 70% of cancer patients will be elderly.

Although some CAM techniques/products could be integrated into cancer care, much more research is needed to confirm their efficacy. Moreover, the wide variety of study designs and types of interventions are an obstacle to reach effective conclusions. Additionally, there is a need to study mechanisms of action of various techniques and products using innovative designs such as research on the effects of CAM on apoptotic and angiogenesis pathways; this may be helpful in understanding tumor development and its progression and applying CAM as a part of personalized medicine to ensure cancer-free and better quality of life. 

CAM is primarily used by cancer patients to relieve disease- and treatment-related side effects. Although many of the symptoms usually subside after treatment, CAM utilization could help maintain a symptom-free and good quality of life during cancer treatment. The word “stress” cannot be over emphasized when associated with cancer experience. Stress reported by cancer patients could potentially alert healthcare providers about the impending negative outcomes of cancer treatments. Most CAM techniques are relatively inexpensive, simple to administer or practice, and encompass the holistic nature of healing. CAM for stress management could restore a patient's sense of control, maintain quality of life, reduce risk of cancer recurrence, and minimize physician visits. The first prescription following a cancer-related visit with a healthcare provider may be for stress management technique/product. Further research on CAM and stress can help healthcare professionals as well as patients with their understanding of the significance of safe use of integrative modes of treatment, better compliance with conventional treatment, improve treatment outcomes and survival, and possibly reduce the risk of recurrence of cancer. Thus, CAM for stress management could be a critical component of cancer care.
